# Clinical Outcome of HIV Viraemic Controllers and Noncontrollers with Normal CD4 Counts Is Exclusively Determined by Antigen-Specific CD8^+^ T-Cell-Mediated HIV Suppression

**DOI:** 10.1371/journal.pone.0118871

**Published:** 2015-03-12

**Authors:** Yada Tansiri, Sarah L. Rowland-Jones, Jintanat Ananworanich, Pokrath Hansasuta

**Affiliations:** 1 Division of Virology, Department of Microbiology, Faculty of Medicine, Chulalongkorn University, Bangkok, Thailand; 2 Nuffield Department of Medicine, NDM Research Building, University of Oxford, Old Road Campus, Headington, OX3 7FZ, Oxford, United Kingdom; 3 Faculty of Medicine, Chulalongkorn University, Bangkok, Thailand; University of Pittsburgh Center for Vaccine Research, UNITED STATES

## Abstract

In this cross-sectional study we evaluated T-cell responses using several assays to determine immune correlates of HIV control that distinguish untreated viraemic controllers (VC) from noncontrollers (NC) with similar CD4 counts. Samples were taken from 65 ART-naïve chronically HIV-infected VC and NC from Thailand with matching CD4 counts in the normal range (>450 cells/μl). We determined HIVp24-specific T-cell responses using standard Interferon-gamma (IFNγ) ELISpot assays, and compared the functional quality of HIVp24-specific CD8^+^ T-cell responses using polychromatic flow cytometry. Finally, *in vitro* HIV suppression assays were performed to evaluate directly the activity of CD8^+^ T cells in HIV control. Autologous CD4^+^ T cells were infected with primary patient-derived HIV isolates and the HIV suppressive activity of CD8^+^ T cells was determined after co-culture, measuring production of HIVp24 Ag by ELISA. The HIVp24-specific T-cell responses of VC and NC could not completely be differentiated through measurement of IFNγ-producing cells using ELISpot assays, nor by the absolute cell numbers of polyfunctional HIVp24-specific CD8^+^ T cells. However, *in vitro* HIV suppression assays showed clear differences between VC and NC. HIV suppressive activity, mediated by either *ex vivo* unstimulated CD8^+^ T cells or HIVp24-specific T-cell lines, was significantly greater using cells from VC than NC cells. Additionally, we were able to demonstrate a significant correlation between the level of HIV suppressive activity mediated by ex vivo unstimulated CD8^+^ T cells and plasma viral load (pVL) (Spearman r = -0.7345, p = 0.0003). This study provides evidence that *in vitro* HIV suppression assays are the most informative in the functional evaluation of CD8^+^ T-cell responses and can distinguish between VC and NC.

## Introduction

Since the first reports of HIV infection over thirty years ago, HIV infection has spread to become a global pandemic [1]. UNAIDS estimate that 35 million individuals were living with HIV throughout the world in 2012. In Thailand, an estimated 0.44 million people are HIV-infected. In the absence of anti-retroviral therapy (ART), there are striking differences in the natural history of HIV infection between individuals: the strongest factor predicting the clinical outcome of HIV infection is the level of plasma HIV load (pVL) [2]. Whilst most infected people fail to control pVL in the chronic phase of HIV infection (noncontrollers, NC), a minority of patients shows evidence of prolonged viral control without ART. Elite controllers (EC), who maintain viral load below the limits of detection, are very rare (estimated at 0.55% in one large study [3]): a slightly larger group (3.34% in the same study) can be classified as viraemic controllers (VC), who maintain pVL below 2,000 copies/ml. Understanding the immune mechanisms that correlate with viral control provides an important opportunity to identify correlates of protective immunity.

For decades in the study of HIV infection, it has been difficult to identify the immune correlates of HIV control precisely. There is considerable evidence to implicate CD8^+^ T cells as playing a key role in HIV control: for example, in one early study, CD8^+^ T cells were shown to be capable of killing HIV-infected CD4^+^ T cells directly [4]. In the macaque model, the control of simian immunodeficiency virus (SIV) infection also correlated with the presence of CD8^+^ T cells [5,6]. Interestingly, several previous studies have indicated that, whilst most HIV proteins can be targeted by CD8^+^ T cells, only the HIV-gag p24-specific (HIVp24) response significantly correlates with reduced pVL levels [7]. Therefore, the objective of this study was to evaluate different characteristics of the HIVp24-specific CD8^+^ T-cell response and to determine their relationship with HIV control amongst HIV-infected VC and NC. We chose to investigate VC as subjects from this category of HIV controllers are more commonly encountered in clinical practice than the very rare EC.

In general, the most common assay used to measure HIV-specific T-cell responses is the measurement of IFNγ-producing T-cells using ELISpot assays: however, several studies have shown that neither the magnitude nor the breadth of the HIV-specific IFNγ-ELISpot response correlates with pVL or other clinical parameters [8,9]. Further studies examined the functional quality of HIV-specific T-cell responses using polychromatic flow cytometry and provided evidence that the ability of CD8^+^ T cells to perform multiple functions, as known as “polyfunctionality”, was an important factor linked with HIV control [10–14]. Other aspects of CTL function that correlate with viral control include preservation of proliferative capacity [12,15] and the presence of higher levels of the cytotoxic factors Granzyme B and Perforin [16,17]. Other studies have suggested that HIV-specific CD8^+^ T-cells from controllers select particular T-cell-receptors [18,19] that mediate high avidity recognition of HIV-infected targets [13,14]. Furthermore, elite controllers (EC) showed higher levels of HIV suppressive activity mediated by CD8^+^ T cells when compared to NC [17,20,21]. However, an evaluation of the T-cell correlates that distinguish between VC and NC has not been reported.

## Materials and Methods

### Ethics statement

This project received approval by Faculty of Medicine, Chulalongkorn University Institutional Review Board (Med Chula IRB), a WHO-certified Ethics Committee, (approval no. 245/53). All volunteers provided written informed consent to participate in this project.

### Subject enrolment

Sixty-five ART-naïve HIV-infected volunteers were recruited from the Anonymous Clinic, Thai Red Cross Society, Bangkok, Thailand. At recruitment, all participants had CD4 counts of more than 450 cells/μl recorded on at least 2 consecutive time-points and were free from Opportunistic Infections (OIs). Participants were categorized as either viraemic controllers (VC) and non-controllers (NC) on the basis of plasma HIV-1 loads (pVL), either below or more than 2,000 copies/ml respectively.

### Preparations of blood samples

Thirty milliliters of EDTA venous peripheral blood were collected for complete blood counts (CBC), pVL, HLA typing, and T cell analyses. Peripheral blood mononuclear cells (PBMC) were isolated by Ficoll-Hypaque density gradient centrifugation (Amersham, Sweden). Viral loads were determined using a quantitative HIV-RNA assay (COBAS AmpliPrep Taqman) with a US-FDA-approved commercial kit in an ISO-15189-certified clinical virology laboratory. CD4 counts were determined by antibody mixture commercial kit (CYTO-STAT triCHROME) in ISO-15189-certified clinical immunology laboratory. HLA-class I type was analysed by PCR-SSOP and PCR-SSP (Proimmune, United Kingdom and BGI Asia-Pacific, Hong Kong).

### IFNγ ELISpot assay

Twenty-three overlapping HIVp24 peptides (OLPs) (20 amino acids long, overlapping by 10 amino acids) and seven previously identified immunodominant epitopes within the HIVp24 protein (accession numbers: JN704002–JN704066) were designed according to a Thai consensus HIV sequence (Mimotope, Australia) [11]. HIV-specific responses were analysed by IFNγ ELISpot as previously described [22]. In brief, 2.5×10^5^ of freshly isolated PBMCs were added to IFNγ-coated 96-well polyvinylidene plate (Millipore, USA) in duplicate for each experiment. PBMC were stimulated with 10 μg/ml HIVp24 peptides for 15 hours at 37°C/5% CO_2_ in a humidified incubator. Phytohemagglutinin (PHA) (Sigma Aldrich, Germany) and recombinant CMVpp65 (rCMVpp65) peptides (Mitilinyl Biotech, USA) were used as positive controls, whilst R10 medium was used as negative control. The spots were counted using an ELISpot reader (Carl-Zeiss, USA) and are presented as Spot Forming Units per million PBMC (SFU/10^6^ PBMCs). Any responses that were at least four times over the average background level after subtraction of negative controls were considered to show positive results.

### Polychromatic flow cytometry

Polychromatic flow cytometry was performed in accordance with standard protocols, as previously described [11,23]. The functional qualities of CD8^+^ T cells were measured using a FACS ARIAII three-laser flow cytometer (Becton Dickinson, USA). At least 1–1.5×10^6^ events were analyzed by FlowJo software (version 10.3) (TreeStar, USA). Analysis of functional T-cell responses by polychromatic flow cytometry using Boolean gating strategy allowed us to investigate 31 distinct phenotypes of HIV p24-specific T cells.

In brief, 10^6^ fresh PBMCs that had been rested overnight were stimulated with 10 μg/ml epitope peptides previously identified as eliciting responses in ELISpot assays. Streptococcus enterotoxin-B (Sigma Aldrich, Germany) and rCMVpp65 protein (Miltinyl Biotech, USA) were used as positive controls. DMSO (Sigma-Aldrich, Germany) and irrelevant peptide were used as negative controls. All cell suspensions were cultured with anti-human CD28, CD49d (Becton Dickinson, USA) Brefeldin-A (Sigma-Aldrich, Germany), and PE-Cy5 anti-CD107a (Biolegend, USA) for 6 hours at 37°C/5% CO_2_ in humidified incubator. Cells were subsequently stained for surface markers including APC-H7 anti-CD3 (Becton Dickinson, USA), Pacific Blue anti-CD8 (Biolegend, USA) for 20 minutes. PBMCs were permeabilized using Cytofix/Cytoperm solution (Becton Dickinson, USA) for 20 minutes at 4°C. For intracellular staining, cells were stained with FITC anti-IL2, APC anti-TNFα, PE-Cy7 anti-IFNγ, and PE anti-MIP1β for 30 minutes at 37°C/5% CO_2_. All intracellular antibodies were purchased from Biolegend except MIP1β (Becton Dickinson). Stained cells were fixed by 1% Paraformaldehyde (Sigma-Aldrich, Germany) and stored at 4°C for detection in following day. Values from negative controls were subtracted from those of CD8^+^ T cells stimulated with HIV peptides, and only responses above the background level were considered as positive results. Absolute numbers of HIV-specific CD8^+^ T cells were calculated using patient CD8 counts.

### Generation of *ex vivo* unstimulated CD8^+^ and CD4^+^ T cells

Both CD8^+^ and CD4^+^ T cells were isolated from PBMC using antibody-coated bead technology. First, the highly enriched CD3^+^ T-cell fraction was negatively isolated from PBMC using Untouched T-cell isolation commercial kit (Invitrogen, USA). CD4^+^ T cells were positively isolated using anti-CD4 microbeads and MS columns (Mitilinyl Biotecs, USA). Unstimulated CD8^+^ T cells were obtained from the negative flow-through fraction.

### Generation of HIV-specific T cell lines

2–5×10^6^ PBMCs were stimulated with 20 μg of HIVp24-specific peptides for an hour at 37°C/5% CO_2_ in a humidified incubator and subsequently cultured in R10 medium with 100 U/ml rIL-2 for 12 days. Expanded HIV-specific T cell lines were rested overnight in R10 medium and subsequently analysed for antigen-specific responses by polychromatic flow cytometry before being used as effector cells in HIV suppression assays.

### Isolation of primary HIV from PBMC

In order to provide CRF01_AE (the dominant strain in our patient population) isolates that could be used in the HIV suppression assays, viral isolation from patient samples was carried out as previously described [24]. Briefly, 1–3 million of HIV-infected donor PBMC were cocultured with 5 million of healthy donor 3-day PHA stimulated PBMC at 37°C/5% CO_2_ for 7 days. Culture supernatants were collected to evaluate the production of newly-generated HIV using HIVp24 Ag ELISA (ZeptoMetrix, USA). HIV-containing supernatants were cryopreserved at -80°C until used. A representative isolate that replicated well in both patient and healthy donor PBMC was selected for use in the suppression assays.

### *In vitro* HIV suppression assay

10^4^ autologous PHA-stimulated CD4^+^ T cells and either unstimulated CD8^+^ T cells or HIV-specific T cell line were co-cultured at an E:T ratio of 1:1 in 100U/ml rIL-2/R10 medium in triplicate or quadruplicate for each experiment. The co-cultured cells were infected with ten-fold diluted primary HIV isolate by spinoculation, except for the negative control wells. At day 3, 7, 10, and 14, the culture supernatants were collected and analysed for levels of HIVp24 Ag using an ELISA assay (ZeptoMetrix, USA) [25]. HIV suppressive activity was calculated as follows: [1—(mean p24 pg/ml in cocultures of CD4^+^:CD8^+^ T cells (1:1) at the same experiment / mean p24 pg/ml in cultures of HIV-infected CD4^+^ T cells alone)] x 100.

### Statistical analysis

Mann-Whitney test, One-way ANOVA (Krusskal-Wallis test), and Spearman’s rank correlation test were performed using GraphPad Prism (version 5.0a). P values of less than 0.05 were considered as statistically significant.

## Results

### HIV controller status was not associated with HLA-B*27/B*57/B*58 alleles

Among 65 HIV-infected volunteers, 19 VC and 46 NC were defined in this study. Demographic data showed that NC had been infected with HIV for a longer time than VC: however this was not statistically significant (Table 1). Median CD4 counts observed in VC did not differ from those seen in NC (681 VS 666 cells/μl). As expected, the median pVL of VC (742 copies/ml) was significantly lower than median pVL of NC (15,606 copies/ml). Several studies have demonstrated strong associations between the presence of HLA-B*27 and certain alleles of the HLA-B*57/58 supertype with improved HIV clinical outcomes in different ethnic groups [26]. In addition, our previous study found a significant association between possession of HLA*B27 alleles and good HIV clinical outcome in Thai subjects, identified by low pVL and high CD4 counts [11]. In this study, however, these protective alleles were not over-represented in VC (36.84%) compared to NC (34.69%). This result indicates that HIV controller status was not associated with the presence of known protective HLA-B alleles in our study population.

**Table 1:** Characteristics of HIV-infected volunteers.

Characteristics	VC (N = 19)	NC (N = 44)
**Female, %**	26.32%	25%
**Male, %**	73.68%	75%
**Heterosexual, %**	26.32%	29.54%
**Homosexual, %**	73.68%	70.46%
**Ages at HIV-1 diagnosis, years**	26 (20–35)	26 (17–54)
**Ages at HIV status characterization, years**	29.5 (23–53)	29 (20–29)
**Year at HIV-1 diagnosis**	2007 (1999–2012)	2009 (2001–2011)
**Year at HIV status characterization**	2011 (2009–2012)	2011 (2009–2012)
**Time at HIV-1 diagnosis, years**	4 (1–12)	4 (1–12)
Frequency of volunteers with HLA-B*27/B*57/B*58, %	36.84%	34.69%
**CD4 counts, cells/μl**	681 (468–1,540)	666 (450–1,045)
**CD8 counts, cells/μl**	944 (409–2,029)	1,096 (500–2,220)
**pVL, copies/ml**	742* (96–2,080)	15,606* (2,472–446,037)

*p value <0.*05*,

*VC = Viraemic controller*, *NC = Noncontrollers*, *pVL = Plasma viral loads*, *HLA = Human leucocyte antigen*, *Data showed medians and range in parentheses*

### Breadth and magnitude of IFNγ-producing T-cell responses against HIVp24

**Fig 1:**
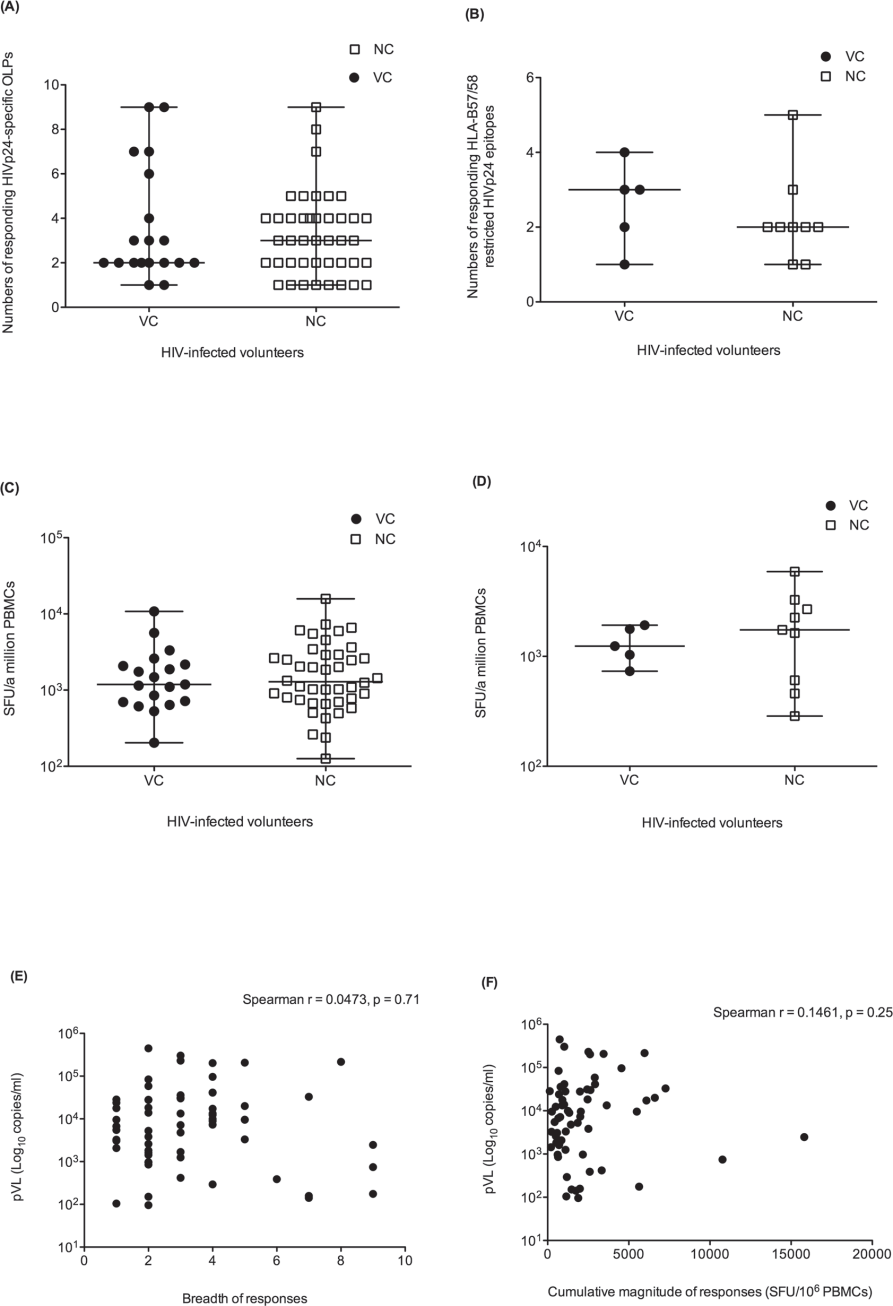
Measurements of IFNγ-producing cell responses using ELISpot assay. **(A)** Breadth of T-cell responses against 23 HIVp24-specific OLPs from VC (N = 19, filled circle) and NC (N = 42, open rectangle) **(B)** Breadth of T-cell responses against HLA-B*57/B*58-restricted epitopes (N = 6) in HLA-B*57/B*58 positive VC (N = 5, filled circle) and NC (N = 9, open rectangle) **(C)** Cumulative magnitude of T-cell responses against 23 HIVp24-specific OLPs in VC (N = 19, filled circle) and NC (N = 42, open rectangle) **(D)** Cumulative magnitude of T-cell responses against HLA-B*57/B*58-restricted epitopes (N = 6) in HLA-B*57/B*58 positive VC (N = 5, filled circle) and NC (N = 9, open rectangle). **(E and F)** Correlation between breadth and cumulative magnitude of T-cell responses against HIVp24-specific OLPs and patient pVL were analyzed by using Spearman’s rank test.

Freshly isolated PBMCs were used for the determination of T-cell responses against 23 HIVp24-specific overlapping peptides (OLPs) using IFNγ ELISpot assays. Most of the HIV-infected volunteers (93.85%) demonstrated responses directed to at least one OLP, except for one NC. Analysis of HIVp24-specific T-cell responses in VC (N = 19) and NC (N = 42) revealed that there were no significant differences in the median breadth of responses between VC and NC (the median number of responding OLP were 2 VS 3) (Fig. 1A). The breadth of responses against 6 HLA-B*57/B*58-restricted epitopes was also similar in VC (N = 5) and NC (N = 9) carrying these alleles (the median number of responding epitopes were 3 VS 2 epitopes) (Fig. 1B). This finding indicated that the breadth of HIVp24-specific T-cell responses, even when directed through “protective” HLA alleles, was not associated with HIV controller status in this study.

For IFNγ-producing T-cell responses against HIVp24, the median cumulative magnitude of responses in VC was not significantly different from those in NC: median cumulative magnitude of responses against HIVp24 OLPs in VC and NC were 1,186 SFU/10^6^ PBMCs (ranging from 204 to 10,776) and 1,289 SFU/10^6^ PBMCs (ranging from 126 to 15,814), respectively (Fig. 1C). Similarly, the median cumulative magnitude of T-cell responses against HLA-B*57/B*58-restricted epitopes in VC did not differ from that in NC (1,240 VS 1,742 SFU/10^6^ PBMCs) (Fig. 1D). Additionally, we were unable to detect any significant correlation between either the breadth or cumulative magnitude of responses and pVL (Fig. 1E and 1F). These findings showed that HIV controller status was not associated with either the breadth or magnitude of IFNγ-producing T-cell responses.

### Absolute cell numbers of polyfunctional HIVp24-specific CD8^+^ T cells were higher in VC than NC

Based on the accumulated results of previous studies and our own findings, it is clear that measuring HIVp24-specific IFNγ-producing T-cell responses by ELISpot may not provide an accurate estimation of T cell-mediated HIV control. It has been previously suggested that the quantity of polyfunctional HIV-specific CD8^+^ T cells may provide a better indication of the robustness of HIV immune control. We previously found that the absolute numbers of polyfunctional HIVp24-specific CD8^+^ T cells were significantly higher in VC than NC, whereas the percentages of polyfunctional cells were not [11]. Taking advantage of the larger sample size and CD4 count matching in this cohort, we revisited the analysis of the functional quality in terms of percentage of cells and absolute cell numbers (calculating from CBC, CD4, and CD8 counts). These phenotypes were also grouped into 5 function groups: 1-, 2-, 3-, 4-, and 5-function group. The groups with 4 or 5 functions were defined as polyfunctional T cells, whilst those with 3-, 2-, and 1-function were termed T cells with limited function. Cumulative absolute cell numbers of 5-, 4-, 3-, 2-, and 1-function HIVp24-specific CD8^+^ T cells were compared between VC (N = 18) and NC (N = 31).

**Fig 2:**
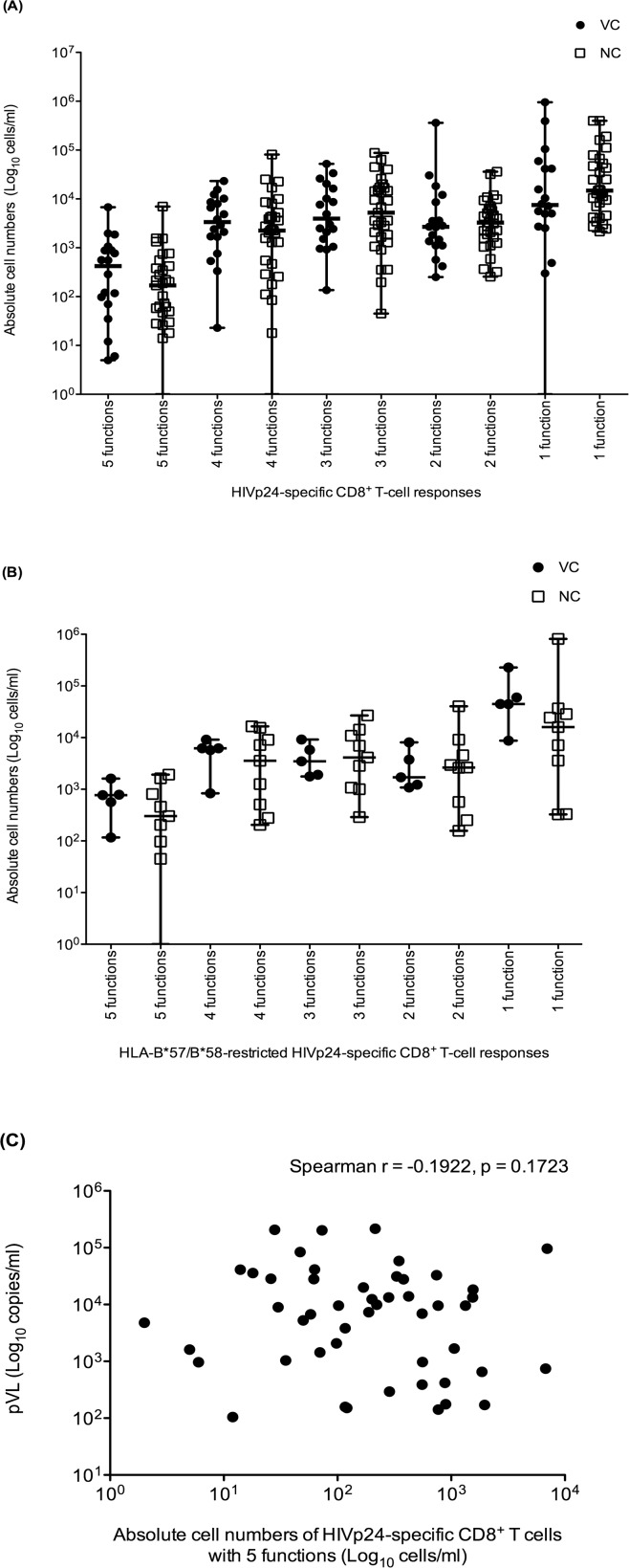
Measurement of functional quality of HIVp24-specific CD8^+^ T cells using polychromatic flow cytometry. **(A)** Functional quality of CD8^+^ T-cell responses against HIVp24-specific OLPs (N = 23) in VC (N = 18, filled circle) and NC (N = 31, open rectangle) **(B)** Functional quality of CD8^+^ T-cell responses against HLA-B*57/B*58-restricted HIVp24-specific epitopes (N = 6) in HLA-B*57/B*58 positive VC (N = 5, filled circle) and NC (N = 9, open rectangle). The Y-axis represents absolute CD8^+^ T cells and the X-axis represents CD8^+^ T cells with 5-, 4-, 3-, 2-, and 1-function in VC and NC. **(C)** The correlation between the absolute cell numbers of 5-functional HIVp24-specific CD8^+^ T cells and pVL was analysed using Spearman’s rank test.

Absolute HIVp24-specific CD8^+^ T cells with the full range of 5 functions were present in higher numbers in VC than NC (median 421 VS 169 cells/ml) but this did not reach statistical significance. Similarly, the 4-function CD8^+^ T cells showed a trend towards higher levels in VC than in NC (3,365 VS 2,258 cells/ml, not significant). On the other hand, T-cell responses with 2 or 3 functions were considerably higher in NC than in VC (again, not significant). Additionally, monofunctional HIVp24-specific CD8^+^ T cells were more frequently detected in NC than in VC. These results show a trend towards the accumulation of polyfunctional CD8^+^ T cells in VC compared with NC but this does not reliably distinguish between these groups. More T cells with limited function, on the other hand, were observed in NC than VC (Fig. 2A).

In addition, the functional qualities of CD8^+^ T-cell responses against classical HLA-restricted epitopes were also determined. We found that the absolute cell numbers of responding polyfunctional T-cells directed against HLA-B*57/B*58-restricted epitopes were greater in VC than in NC expressing these HLA alleles (Fig. 2B). However, there was no significant correlation between the absolute numbers of CD8^+^ T cells with the full 5 functions and pVL (Fig. 2C).

### *Ex vivo* unstimulated CD8^+^ T cells of VC effectively inhibit HIV replication

**Fig 3:**
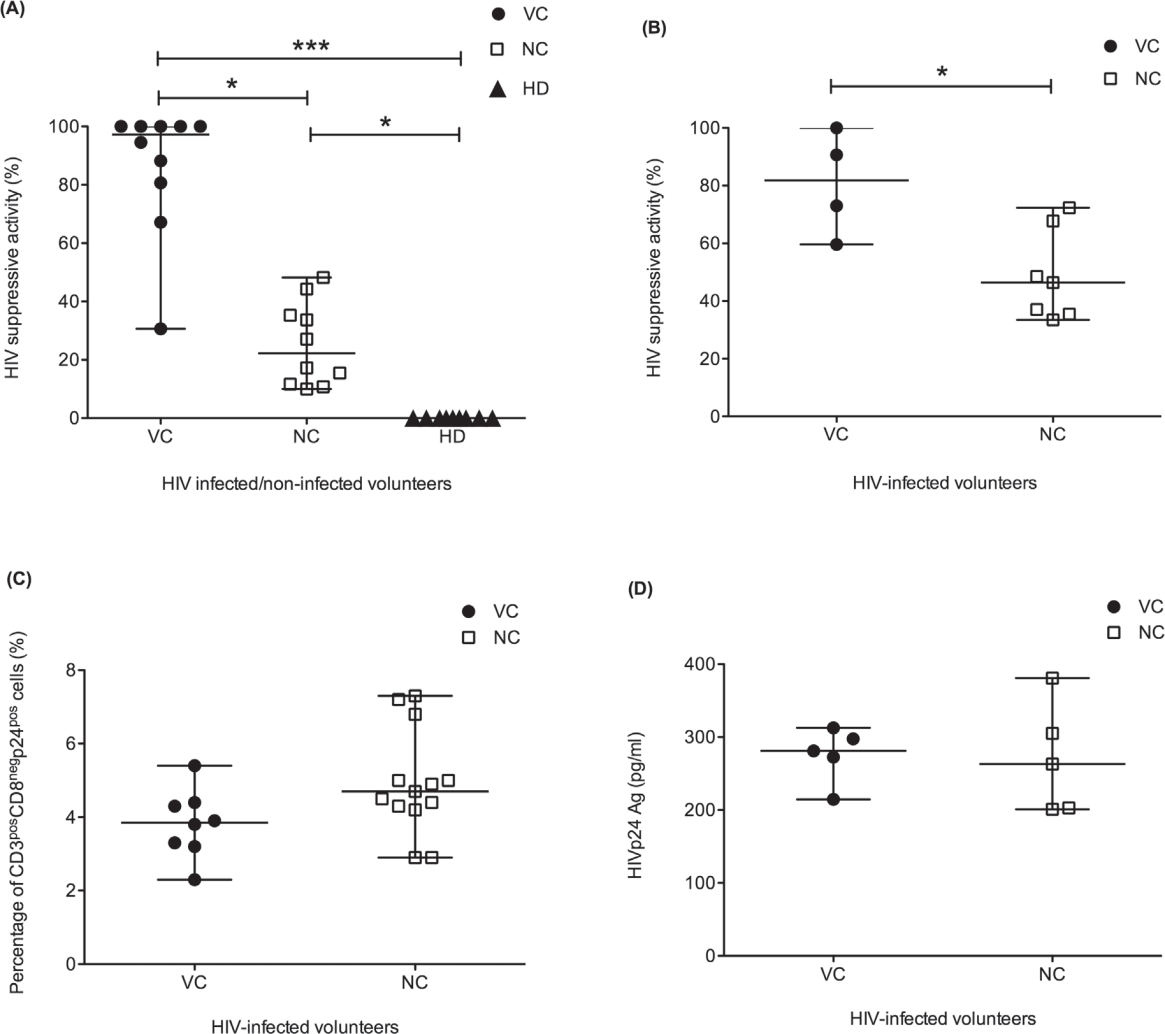
*In vitro* HIV suppression assay. **(A)** *In vitro* HIV suppression assay mediated by *ex vivo* unstimulated CD8^+^ T cells in VC (N = 10, filled circle), NC (N = 10, open rectangle), and healthy volunteers (HD, N = 9, filled triangle) cocultured with autologous HIV-infected CD4^+^ T cells at E:T ratio of 1:1 at day 7. **(B)** *In vitro* HIV suppression assay mediated by HIVp24-specific T cell lines from VC (N = 4, filled circle) and NC (N = 7, open rectangle) cocultured with autologous HIV-infected CD4^+^ T cells at E:T ratio of 1:1 at day 3. **(C)** Percentages of *ex vivo* HIV-infected CD4^+^ T cells (CD3^pos^CD8^neg^P24^pos^) in VC (N = 8, filled circle) and NC (N = 13, open rectangle) **(D)** Production of newly-generated HIV from CD4^+^ T cells from VC (N = 5, filled circle) and NC (N = 5, open rectangle) at day 7. The Y-axis depicts the concentration of HIVp24 Ag (pg/ml) from ELISA assays. *p value <0.05 and ***p value<0.001.

In order to demonstrate direct T cell-mediated HIV control, we determined the ability of CD8^+^ T cells to inhibit HIV replication using an *in vitro* HIV suppression assay. In a modification of the methods used in other studies, we used a primary HIV isolate from an HIV-infected volunteer presumed to be infected with CRF01_AE in our experiments, rather than laboratory-adapted HIV strains (which are predominantly clade B). At CD8:CD4 ratio of 1:1, *ex vivo* unstimulated CD8^+^ T cells from either VC (N = 10) or NC (N = 10) were able to inhibit HIV replication (Fig. 3A), albeit at different potency. Significantly higher in HIV suppressive activity by unstimulated CD8^+^ T cells *ex vivo* was demonstrated in VC (97.27%) when compared with CD8^+^ T cells from NC (22.22%) (p<0.05). No HIV suppressive activity could be detected when CD8^+^ T cells from healthy donors were used (n = 9), suggesting that HIV suppression in these assays was mediated by HIV-specific CD8^+^ T cells (Fig. 3A).

To exclude the possibility that these results might reflect a higher frequency of HIV-infected CD4^+^ T cells in NC we evaluated the frequency of infected cells *ex vivo* using intracellular p24 staining. Comparable levels were seen in VC (n = 8) and NC (n = 13) (Fig. 3C). In addition, to reduce the chance of cells from VC and NC exhibiting different levels of susceptibility to primary HIV isolates, the production of newly-generated HIV from VC (N = 5) and NC (N = 5) cells was compared using p24 ELISA assay and found to be similar (Fig. 3D).

### Significantly greater HIV suppressive activity by HIVp24-specific T cell lines from VC compared to T cell lines from NC

To confirm that the HIV suppressive activity previously detected was mediated by HIV-specific CD8^+^ T cells, the effector cells were replaced with HIVp24-specific T cell lines. The HIV suppressive activity of lines from both VC (n = 4) and NC (n = 7) reached 100% at day 7 (data not shown). However, at by day 3 there was a clear difference between the HIV suppressive activities of VC and NC T cell lines (p = 0.0242). At day 3, the median percentages of HIV suppressive activity were 81.87% (ranging from 59.62% to 100%) for VC and 46.37% (ranging from 33.51% to 72.33%) for NC (Fig. 3B). These results showed that HIV-specific T cell lines derived from VC had significantly better HIV suppressive activity than T cell lines grown from NC.

### Efficacy of HIV suppressive activity is dependent on the quantity of effector cells

**Fig 4:**
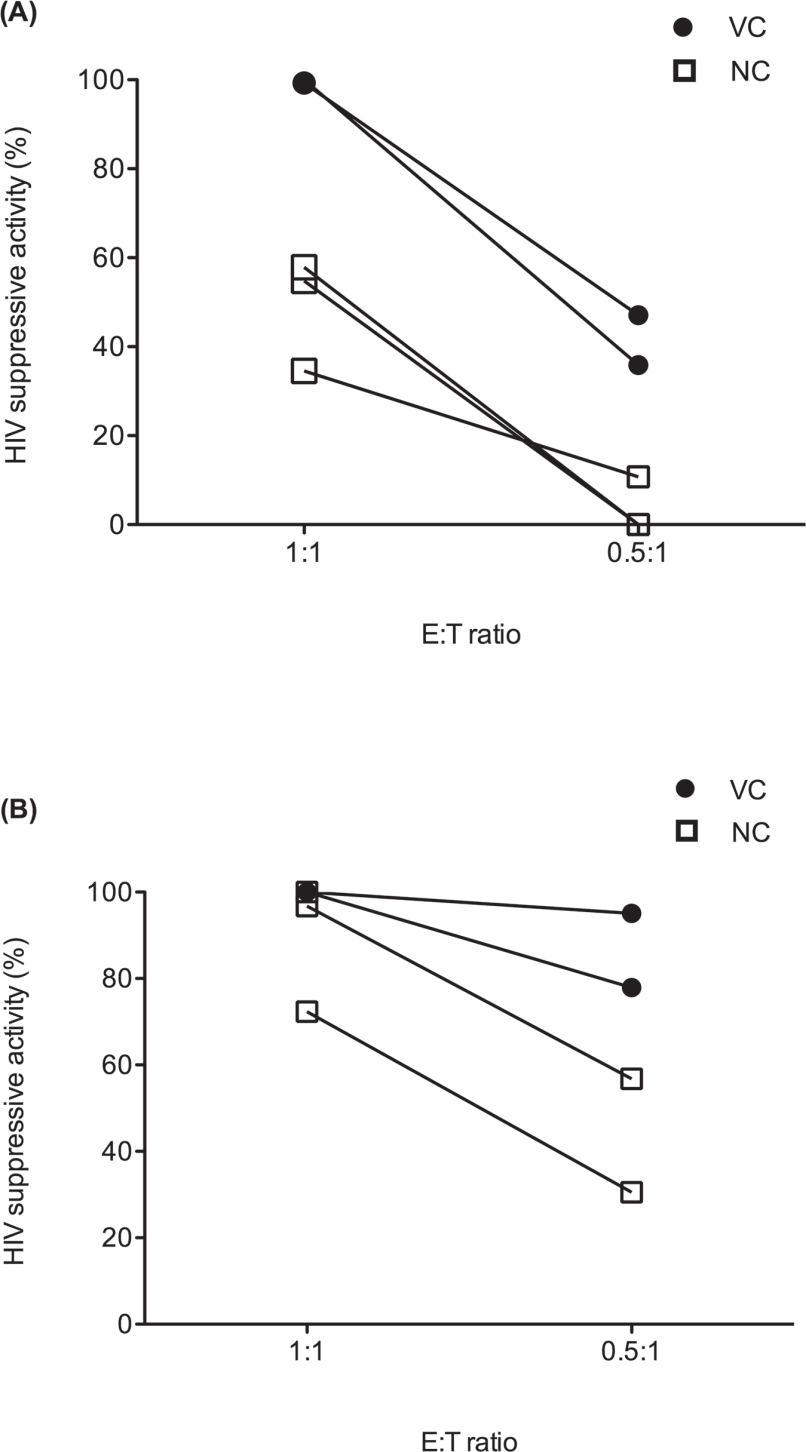
*In vitro* HIV suppression assay in various E:T ratios. **(A)** *In vitro* HIV suppression assay mediated by *ex vivo* unstimulated CD8^+^ T cells in culture with autologous CD4^+^ T cells at various E:T ratios of 1:1 and 0.5:1 in VC (N = 2, filled circle) and NC (N = 3, open rectangle). **(B)** *In vitro* HIV suppression assay mediated by HIVp24-specific T cell lines at various E:T ratios of 1:1 and 0.5:1 in VC (N = 2, filled circle) and NC (N = 2, open rectangle).

To investigate the quantity of effector cells required for HIV suppressive activity, the E:T ratio was decreased from 1:1 to 0.5:1. At an E:T ratio of 1:1, the HIV suppressive activity mediated by unstimulated CD8^+^ T cells was higher in VC (N = 2) than NC (n = 3), with medians of 99.65% and 54.80% on day 7, respectively (Fig. 4A). Furthermore, at an E:T ratio of 0.5:1, HIV suppressive activity for VC was 41.51%, whereas no suppressive activity could be detected from NC on day 7 (Fig. 4A). As for HIV suppressive activity mediated by HIV p24-specific CD8^+^ T cells, T cell lines from VC (n = 2) could completely suppress HIV replication at an E:T ratio of 1:1 on day 3, whereas the HIV suppressive activity of NC lines was lower, at 84.55%. Meanwhile, at an E:T ratio of 0.5:1, HIV suppressive activities mediated by HIV p24-specific CD8^+^ T cells were also greater in VC when compared with NC (Fig. 4B). These data showed that decreasing the quantity of effector cells from either unstimulated CD8^+^ T cells or HIV p24-specific T cell lines diminished the *in vitro* HIV suppressive activity. In both settings, a greater loss of HIV suppressive activity mediated by HIV-specific T cell lines was observed in NC compared with VC.

### Significant correlation between HIV suppressive activity and pVL

**Fig 5:**
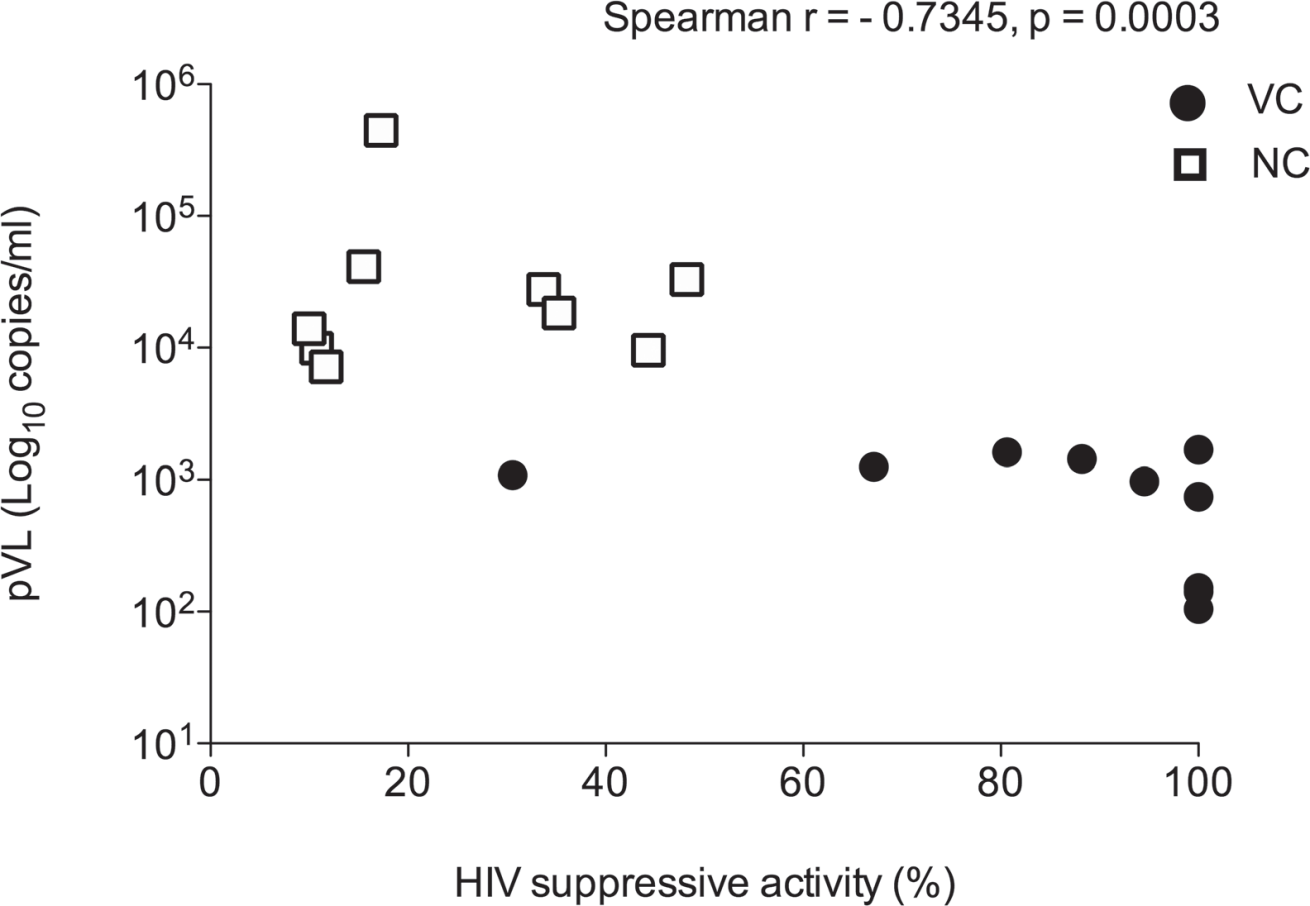
Correlation between HIV suppressive activity and pVL. *In vitro* HIV suppression assay mediated by *ex vivo* unstimulated CD8^+^ T cells at E:T ratio of 1:1 from HIV-infected volunteers (N = 19) were shown on the X-axis as percentages of HIV suppression. The pVL at the same time-point as the HIV suppression assay are presented on the Y-axis. Each symbol represents a single HIV-infected volunteer. Filled circles and open rectangles represent viraemic controllers and noncontrollers, respectively. Spearman’s rank test was analyzed as the statistic.

Taken together these data clearly demonstrate a strong relationship between CD8^+^ T-cell-mediated suppression of HIV *in vitro* and clinical evidence of viral suppression. VC had superior CD8^+^ T-cell responses with better ability to inhibit HIV replication compared to NC. To provide further support, we evaluated the correlation between *in vitro* HIV suppressive activity and *in vivo* control of pVL. Amongst 19 HIV-infected volunteers, the level of HIV suppressive activity mediated by unstimulated CD8^+^ T cells was evaluated in relation to pVL (Fig. 5). A highly significant correlation between *in vitro* HIV suppressive activity and pVL was observed (Spearman r = -0.7345, p = 0.0003). This result provides strong evidence that CD8^+^ T cells contribute to HIV control.

## Discussion

This study has demonstrated the value of measuring distinct aspects of the CD8^+^ T-cell responses against HIV in relation to clinical outcome and viral control. In our patient cohort, all of whom were at a relatively early stage of HIV infection and presented with a CD4 count in the normal range, the *in vitro* HIV suppression assay showed a much better correlation with pVL and clinical status than the results of either standard IFNγ ELISpot assays or flow cytometric polyfunctional assessment. The superiority of HIV-specific CD8^+^ T cells that potently inhibit HIV replication *in vitro* appears to provide an accurate reflection of the clinical status of viraemic controllers (VC), who naturally control pVL *in vivo* to less than 2,000 copies/ml. These results provide new insights into the evaluation of T-cell responses and their relationship to HIV control.

Despite a wealth of evidence to support an important role of CD8^+^ T cells in HIV control, the precise immune correlates of protective immunity in HIV infection are still poorly defined. T cells are believed to play an essential role in the small proportion of HIV-infected individuals with good clinical outcome [8,13–18,20]. Several previous studies have shown that T-cell responses in controllers exhibit higher quality when compared to their noncontrollers counterparts: however most studies have enrolled NC or progressors at relatively advanced stages of HIV infection when it is hardly surprising that these NC/progressors did not demonstrate favourable T-cell responses [10–12]. In order to avoid this important potential confounding factor, we enrolled the subjects in our study with similar baseline CD4 counts. In addition, as far as we can tell, both groups were likely to have become HIV-infected in a similar frame (Table 1), so there is no major discrepancy in disease stage between our study groups other than plasma viral loads.

Many publications have reported strong associations between certain HLA alleles, predominantly B alleles, and good clinical outcome [26–28]. In the largest reported genetic study of HIV controllers, the major association detected was with the class I HLA region on chromosome 6, mapping to a few amino acids in the peptide-binding grooves [29]. Surprisingly, we did not find any enrichment of the well-described protective alleles in our VC (Table 1). Similarly, HLA-B*51 allele, which has recently been described as a protective allele in Han Chinese [30], was not detected at all in our subjects. It is probable that other, as yet un-described, protective HLA alleles may play a role in controlling HIV replication in Thai individuals. Previously, the common Thai class I HLA allele, HLA-A*11:01 was proposed to be protective in highly exposed persistently seronegative persons in Thailand [31]: however this HLA allele was not enriched in our VC group. In fact, no HLA alleles were associated with viraemic controller status in our study: this may reflect the relatively small sample size. In sharp contrast to previous studies, volunteers in our study expressing the well-characterised protective HLA alleles tended to have higher pVL, although this was not statistically significant.

Antigen-specific T-cell responses have been shown to correlate with protective immunity in a number of persistent viral infections [5–8,14,32–35]. By analogy to antiviral drugs, T-cell responses against different viral targets may confer differential ability to control viral replication [36]. Indeed, several studies have demonstrated a relationship between T-cell responses to gag [7,10,21,27,37,38], particularly p24 [7,39], and control of HIV replication. We therefore elected to focus on HIV p24-specific T-cell responses in our subjects in order to characterise protective immunity in HIV infection. In order to ensure the quality of our ELISpot assays, we always used fresh PBMC in our experiments; and to avoid antigen mismatch, we synthesized OLP based on recently-circulating Thai HIV strains [11]. However, our ELISpot experiments failed to show any difference in T-cell responses between VC and NC: in fact, NC showed a trend towards a greater breadth of response. As others have shown [11,40], production of IFNγ alone is unlikely to be a good marker of the ability to control HIV replication. Several previously-published studies, including ours, have demonstrated that HIV-infected individuals with good clinical outcomes and/or with protective HLA alleles had polyfunctional T cells [10–13]. Our current work, on the other hand, did not reproduce this result. Although VC showed a trend towards having greater absolute numbers of HIV p24-specific polyfunctional T cells, these findings failed to reach statistical significance. The discrepancy between previous findings and ours may reflect the fact that we compared T-cell responses between two similarly immunocompetent (as estimated by CD4 counts) groups. HIV-specific T cells may lose their polyfunctional capacity as CD4 counts fall in late HIV infection. The lack of adequate CD4 T-cell help may lead to typical progressor/NC volunteers having less functional T-cell responses, as shown in previous studies [11]. In contrast, our NC were relatively immunocompetent, as reflected by their high CD4 counts, and hence were able to mount polyfunctional T-cell responses against HIV infection [41], narrowing the gap between VC and NC in terms of T-cell polyfunctionality in this study.

Reliable estimates of immune correlates of protection are essential as a read-out for HIV vaccine trials. ELISpot and polyfunctional assays are, in our opinion, unlikely to be the ideal methods to determine the potential efficacy of candidate HIV vaccines. Recently, several groups have shown that CD8^+^ T cells studied *ex vivo* could inhibit HIV replication *in vitro* [19–21,42,43]. This HIV suppression ability was more pronounced in infected individuals with good clinical outcome, but in general these studies have tended to compare a group with high CD4 counts with typical progressors who had lower CD4 counts. As it could be reasoned that an ability to suppress HIV replication would depend on CD4 help, we compared the HIV suppressive ability of VC and NC who had similar baseline CD4 counts. Interestingly, unlike polyfunctional responses, *ex vivo* CD8^+^ T-cell-mediated suppression ability of VC was significantly higher than that of NC. Our study demonstrated significantly higher HIV suppressive activity mediated by unstimulated CD8^+^ T cells from viraemic controllers (pVL <2,000 copies/ml) compared with cells from progressors/noncontrollers (pVL >2,000 copies/ml).

Our results are consistent with previous cross-sectional studies of HIV-1 clade-B-infected elite controllers (pVL <50 copies/ml), which revealed substantial HIV suppressive activity using unstimulated CD8^+^ T cells compared with that seen in noncontrollers (pVL >50 copies/ml) [17,20,21]. In a further study using a CD8^+^ T cell-based HIV suppression assay, viraemic controllers (pVL <5,000 copies/ml) showed significantly greater HIV inhibition than non-controllers (pVL >5,000 copies/ml) [42]. Chinese subjects with primary HIV infection were also recruited in this study to examine the predictive value of HIV suppression assays, and the degree of HIV suppression was shown to correlate strongly with slower CD4 decline. In this study, there was no relationship between viral suppression and the IFNγ response, similar to our findings. However, other groups have reported a strong correlation between suppressive activity and the frequency of virus-specific T-cells, particularly when HIV-gag specific T cells were analysed [20,21]. This may reflect differences in the infecting clade of HIV-1 and the immunogenetic background of the patients studied. Whilst the relationship between gag-specific CTL responses and HIV suppression was seen in a largely clade B-infected Caucasian cohort enriched for HLA-B*57 and HLA-B*27, our study enrolled Thai individuals likely to be predominantly infected with CRF01_AE, who lacked these protective alleles. Intriguingly a recent report has highlighted the absence of HIV suppressive activity in subjects studied very early in the course of HIV infection [41], suggesting that the ability of CD8+ T-cells to suppress viral replication is acquired at a later stage of infection.

The suppression of viral replication by HIVp24-specific T cell lines implies that the CD8^+^ T cells in our *ex vivo* experiments were HIV-specific. Interestingly, the ability to suppress HIV replication showed a strong and highly significant inverse relationship with plasma HIV-RNA. Although the previous reports described above differ in study design, the nature of the subjects enrolled and the assay techniques employed, our results are consistent with the observations that greater HIV-suppressive ability is seen in HIV-infected individuals with a favourable clinical outcome [19–21,42,43]. These data together provide strong supporting evidence for the importance of CD8^+^ T-cell responses in the control of HIV viraemia.

We adopted the methodology previously described by Saez-Cirión with a slight modification [25]. In essence, we did not use laboratory strains due to the evidence that the ability to suppress HIV may not always be cross-reactive between different viral clades [21]. Instead, we isolated primary isolates from Thai subjects who were infected with HIV subtype CRF01_AE. The viruses we isolated showed a similar degree of *in vitro* replicative fitness regardless of whether they came from VC or NC (unpublished data). Similarly, spontaneous production of HIVp24 antigen was not higher in NC than VC, consistent with previous reports [43]. Taken together, these findings suggest that impaired viral replicative fitness is not an essential element in maintaining viraemic controller status.

The superiority of HIV inhibition in controllers described in most published studies could potentially be explained by presentation of particularly vulnerable epitopes presented by protective HLA class I alleles. These epitope targets have been extensively investigated and it is probable that effective control of HIV replication mediated through these epitopes is a consequence of either the highly-sensitive recognition of infected cells presenting the wild-type epitope or the reduction of viral fitness that occurs after epitope “escape” mutations. A good example of this phenomenon would be the HLA-B*27-restricted ‘KK10’ epitope in HIV p24 gag [19], where “escape” mutations are associated with reduced HIV replication [39,44]. T-cell responses against this epitope are very dominant and, in most subjects bearing this allele, no critical epitope mutations can be detected. Our HLA-B*27 volunteers, except those with HLA-B*27:06, also make a dominant response against this well-characterised epitope. With the exception of substitutions between L/M at position 6 of the 10-mer epitope, for which cross-reactive recognition is usually seen, we did not see any other mutations within this epitope (unpublished data). Our subjects with HLA-B*27 and other protective HLA-B alleles did not suppress HIV replication better than those without protective alleles, in contrast to the findings of other groups studying HLA-B*2705-restricted responses in Caucasians [13,14]. We cannot exclude the possibility that there might be other HLA-B*27-restricted protective epitope(s) which could have mutated in our HIV-infected volunteers. Nor can we rule out that HIV-infected cells from Thai individuals may express fewer HLA-B*27-KK10 complexes than do Caucasians, or be subject to KK10-specific CTL of lower functional avidity.

Since our research design was cross-sectional, we could not draw any conclusions about the effect of HIV suppression on CD4 decline or survival advantage. However, Dorrell and her group elegantly demonstrated that greater T-cell-mediated HIV suppression correlated well with survival advantage and inversely correlated with CD4 loss [42]. It will be interesting to see if the long-term clinical outcome of HIV-infected Thai individuals could be predicted by HIV suppressive activity. Our findings imply that the evaluation of HIV vaccine responses using ELISpot or polychromatic cytokine/chemokine assays may not accurately reflect a protective immune response, whereas the *in vitro* HIV suppression assay is, in our opinion, significantly more useful to differentiate between vaccinees who have potentially developed protective immunity and those who have not. However, this method is expensive, time-consuming and requires the operation of skillful scientists. Further efforts to make assays of HIV suppression more practical and reliable for large-scale vaccine trials should be encouraged [45].
